# Association between the canal filling ratio and bone resorption in trabecular metal stems in reverse total shoulder arthroplasty: a radiographic analysis using tomosynthesis

**DOI:** 10.1016/j.jseint.2024.05.010

**Published:** 2024-05-30

**Authors:** Kazumasa Takayama, Hiromu Ito

**Affiliations:** Department of Orthopaedics, Kurashiki Central Hospital, Japan

**Keywords:** Osteopenia with cortical narrowing, CNO, Trabecular metal, Canal filling ratio, Reverse shoulder, Bone resorption, Tomosynthesis

## Abstract

**Background:**

Several factors affect the incidence of osteopenia with cortical narrowing (CNO) in reverse shoulder arthroplasty. This study aimed to compare the incidence of CNO with different fixation methods (cemented or cementless) using a single implant (trabecular metal humeral stem) evaluated using tomosynthesis and to analyze the factors affecting the incidence of CNO for cementless stem fixation.

**Methods:**

A total of 109 patients (cementless: 75 cases; cemented: 34 cases) who underwent reverse total shoulder arthroplasty were included in this study. The patients were divided into 2 groups (cementless or cemented), and the incidence of CNO was compared. In addition, patients in the cementless group were divided into 2 groups (canal filling ratio [CFR] of ≥ 0.7 or < 0.7), the incidence of CNO was compared, and the Cramer’s coefficient of association between CNO and CFR > 0.7 (and 0.8) was calculated.

**Results:**

No significant difference was observed in the incidence of CNO between the cementless and cemented groups (7/75 vs. 3/35, *P* value = 1.0). The association between CNO and the CFR using Cramer’s coefficient of association showed that there were few correlations (coefficient: 0.14, *P* value = .59).

**Conclusion:**

Cementless reverse total shoulder arthroplasty with a trabecular metal stem has a similar low incidence of CNO as cemented fixation, and the incidence of CNO with a trabecular metal stem was lower than that reported in previous studies. A CFR > 0.7 was not associated with the incidence of CNO.

Reverse total shoulder arthroplasty (RTSA) has been performed successfully in patients with irreparable rotator cuff tears and shoulder pseudo-paralysis. Historically, good clinical results have been reported with the use of cemented humeral stems,^30^^,^^47^ while the use of cementless implants has been the preferred choice in recent years because of concerns regarding difficulty in removal during revision surgery, thrombosis, technical requirements, and prolonged operative time. Recently, however, there have been several reports of osteopenia with cortical narrowing (CNO) around the humerus with cementless stems, which is thought to be caused by stress shielding. Bone resorption around the humeral stems is not only a potential cause of loosening but can also make revision difficult. Factors thought to be associated with CNO include sex,^26^^,^^41^ long stem length,^7^^,^^9^^,^^44^ type of surface coating,^25^^,^^26^^,^^42^^,^^44^ location of the coating,^25^^,^^36^^,^^48^ stem geometry,^2^^,^^11^^,^^37^ and a high canal filling ratio^7^^,^^42^^,^^48^^,^^49^ (CFR). A strong correlation with CNO has been reported, especially when the CFR exceeds 0.7.^42^ On the other hand, cemented stems have been reported to cause less CNO than cementless stems.^7^ This study used either a single implant (trabecular metal humeral stem teres minor [TM] stem; Zimmer Biomet, Warsaw, IN, USA) with cemented or cementless fixation. To the best of our knowledge, few studies have evaluated the incidence of CNO in RTSA with TM stems.^26^

This study aimed to compare the incidence of CNO between cemented and cementless fixation methods using tomosynthesis (SONIALVISION G4; Shimadzu Corp., Kyoto, Japan). Additionally, our results were compared with those of previous studies to analyze the factors affecting the incidence of CNO with respect to cementless humeral stem fixation. We hypothesized that cementless RTSA with the TM stem would have a similarly low incidence of CNO as that in cemented fixation and that the incidence of CNO with the TM stem would be lower than that in previous studies.

## Materials and methods

### Study design and patient selection

This study was a retrospective review of prospectively collected data of patients who underwent RTSA performed by a single surgeon in a single center between October 2014 and January 2021.

Written informed consent was obtained from all patients before enrolment into this study, and the study was approved by the ethics committee of our hospital. The inclusion criterion for RTSA was irreparable rotator cuff tears or cuff tear arthropathy with failed conservative treatment supervised by physiotherapists. All patients were classified as grades 2-5 according to the Hamada classification. Patients with shoulder joint infections or neurological disorders were excluded due to no indication of RTSA. Two types of prostheses were used for RTSA: (1) the Trabecular Metal Reverse Shoulder System (Zimmer Biomet, Warsaw, IN, USA), which was used for humeral stems and baseplates from October 2014 to October 2018 and (2) the Trabecular Metal Reverse Shoulder System for humeral stems combined with the Comprehensive Shoulder System (Zimmer Biomet, Warsaw, IN, USA) from November 2018 to July 2021. Patients on steroids induced osteopenia, cortical thickness ≤ 2 mm at the lateral aspect of the humerus,^55^ or those with torsional instability at the time of the implant placement were indicated for cement fixation.

In total, 119 patients underwent primary RTSA and were followed up for > 2 years. Six patients died due to unrelated causes after the surgery, and we failed to obtain their consent for this study. One patient with prosthesis loosening (TM glenoid) that occurred 6 months postoperatively was excluded. Three patients were lost to follow-up and did not undergo tomosynthesis. In total, 109 patients (cementless, 75; cemented, 34) were included in this study. All patients were followed up at 2, 3, 4, 5, 6, 8, 10, and 12 months postoperatively, and every 12 months thereafter. The mean observation period was 56 (24-101) months and the mean age was 79.2 (66-88) years.

### Radiographical and physical examination assessments

**Figure 1:**
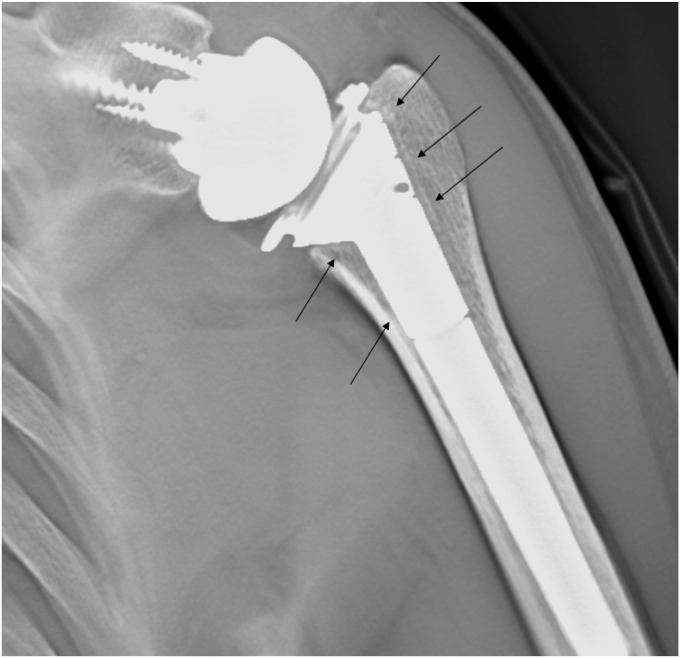
Tomosynthesis with T-smart can reduce the influence of metallic artifacts. *Black arrow*: spot welds.

All clinical data were collected from medical records. The primary aim of this study was to investigate the occurrence of CNO around the humeral stem using plain roentgenography and tomosynthesis in the coronal view with T-smart (Tomosynthesis-Shimadzu Metal Artifact Reduction Technology). Tomosynthesis is a technique to obtain high-resolution tomographic images while irradiating the sample at certain angles, while T-smart is an application that can reduce metallic artifacts (Fig. 1). In plain radiographs, the true anterior-posterior view of the glenohumeral joint is obtained with the patient in a standing position using Radnext 50 (Fujifilm, Tokyo, Japan, distance: 120 cm, antiscatter grid: 8:1). Images were analyzed using the application, Console Advance, Fujifilm, Tokyo, Japan.

**Figure 2:**
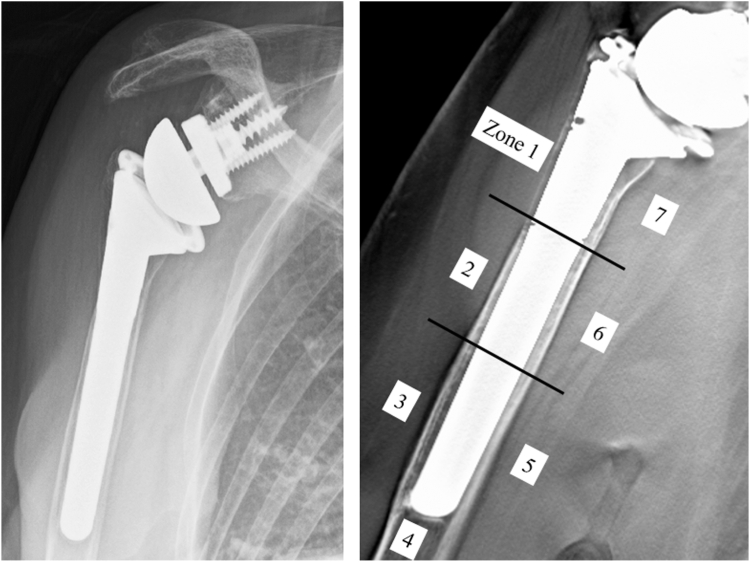
Osteopenia with cortical narrowing were recorded according to the zones where they were located.

**Figure 3:**
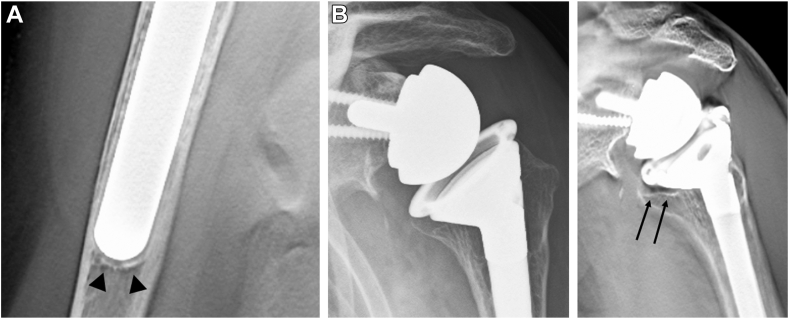
(**A**) Condensation line in zone 4. (**B**) The plain radiograph does not show the osteolysis, but the tomosynthesis clearly shows the osteolysis. *Arrow*: osteolysis.

**Figure 4:**
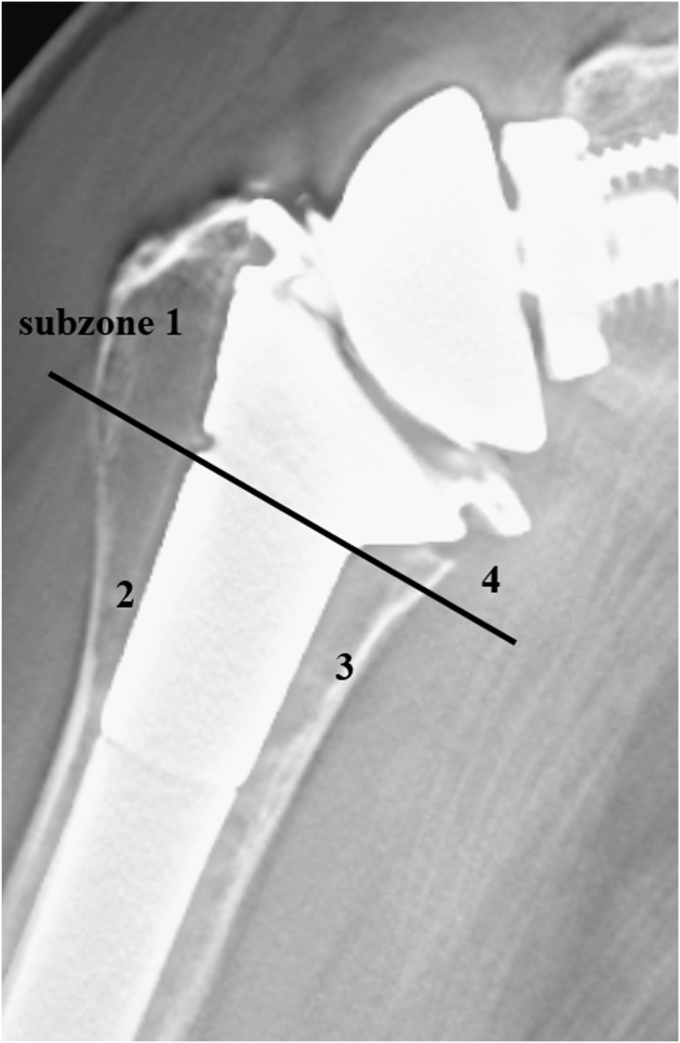
The sites of spot welds around the surface coating area were recorded according to the subzones 1-4.

The sites of osteopenia with CNO were recorded according to the location of the zones (Fig. 2). CNO was graded according to the previous study; grade 0, no bone resorption; grade 1, decrease in the cortical bone density; grade 2, thinning of the cortical bone comprising less than one-half of the original thickness; grade 3, thinning of the cortical bone comprising more than one-half of the original thickness; and grade 4, complete disappearance of the cortical bone.^25^ In addition, other symptoms of stress shielding, such as pedestal sign and condensation line (CL),^32^ (Fig. 3*A*) were recorded where they were located. In addition, osteolysis (OL) around the prosthesis was evaluated (Fig. 3*B*). Bone incorporation between the bone and prosthesis was defined as a confirmation of spot welds (SW) connecting the porous area and bone in more than 2 zones without a radiolucent line.^17^^,^^21^ The sites of SW around the surface coating area were recorded according to the subzones where they were located (Fig. 4). At the last follow-up, all patients underwent tomosynthesis.

**Figure 5:**
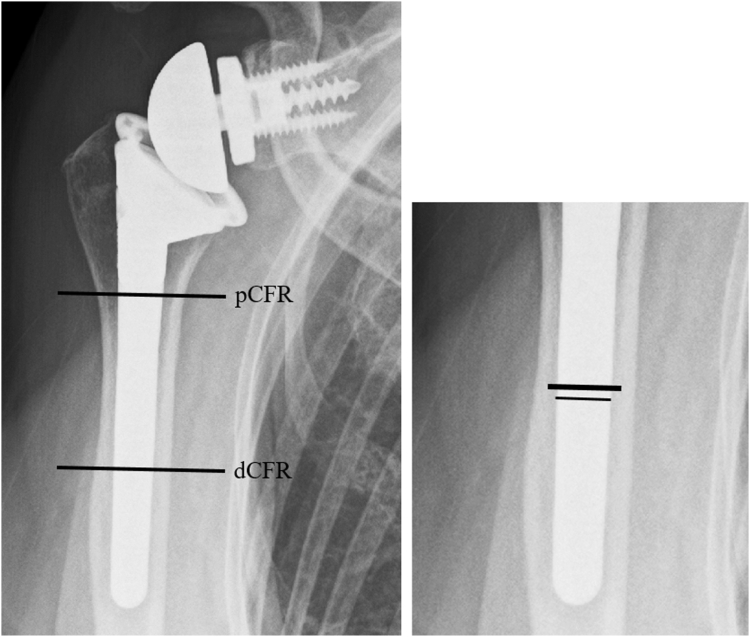
The canal filling ratio was calculated at the proximal and distal third of the humerus using plain radiographs immediately after surgery.

Traditionally, implant loosening was defined as radiolucent lines with a width > 2 mm.^31^^,^^57^ However, 16.3% of implants without loosening on plain radiographs and computed tomography (CT) have been reported to have implant loosening,^40^ suggesting that the traditional criteria failed to detect loose implants. Furthermore, tomosynthesis had been demonstrated to have a higher sensitivity than CT in detecting radiolucent lines < 1.2 mm.^52^ Hence, in this study, stem loosening was defined as the presence of at least 1-mm radiolucency^50^ around the prosthesis in more than 2 zones, as evaluated using tomosynthesis, or the incidence of prosthesis migration (including stem sinking, or valgus and varus angulation > 5°) between the radiographs obtained immediately after surgery and the radiographs acquired at the final follow-up.^12^^,^^30^ The CFR was calculated at the proximal and distal third of the humerus using plain radiographs immediately after surgery^36^ (Fig. 5). The reason why we calculated CFR at the proximal third as well as distal third of prosthesis (dCFR) was that cortical contact at the proximal third may be a factor that affected the bone absorption of tuberosity. A previous study reported that CFR ≥ 0.7 and ≥ 0.8 for anatomic arthroplasty and reverse arthroplasty, respectively, was a critical factor that affected CNO.^42^ Therefore, patients with cementless fixation were divided into 2 groups (dCFR ≥ 0.7 or dCFR < 0.7 and dCFR ≥ 0.8 or dCFR < 0.8), and the incidence of stress shielding symptoms, especially in CNO, was investigated. The presence of varus or valgus angulation of > 5° relative to the bone axis was evaluated using radiographs obtained immediately after surgery.

Moreover, scapular notching was evaluated according to the Sirveaux et al classification.^47^ Two independent trained surgeons not affiliated with the surgery evaluated bone incorporation and stem loosening in each group (cementless or cemented). Inter-rater reliability was examined using Cohen’s kappa statistics.

Shoulder range of motion (ROM), including flexion, abduction, external rotation, and internal rotation, was assessed preoperatively and at the final follow-up visit. Except for internal rotation, the ROM was measured using a goniometer. For the evaluation of internal rotation, the highest vertebral level at which the tip of the thumb could reach was converted to numerals, from the thigh (1 point) to the level of the first thoracic vertebra (20 points).^51^ Furthermore, the American Shoulder and Elbow Surgeons (ASES) score was evaluated preoperatively and at the time of final follow-up, and complications related to RTSA were also evaluated.

### Operative technique

The standard deltopectoral approach was adopted with patients in a beach chair position. If the subscapularis tendon remained intact, it was peeled off. In the Trabecular Metal Reverse Shoulder System, a 15-mm center post of the baseplate and a 36-mm diameter glenosphere were used in all patients.

The Comprehensive Shoulder System (Comprehensive Shoulder System; Zimmer Biomet, Warsaw, IN, USA) used a glenoid and a glenosphere of diameters 25 mm and 36 mm, respectively, on all patients. When preparing the footprint where the baseplate was placed, reaming was performed until the cancellous bone in the lower part of the glenoid was exposed. Drilling at the area of osteosclerotic change was performed using a 1.5-mm Kirschner wire to refresh the footprint.

All patients were implanted with the same humeral prosthesis (Trabecular Metal Reverse Shoulder System; Zimmer Biomet, Warsaw, IN, USA) and positioned at 10° retroversion. In the cementless fixation group, the cancellous bone harvested from the resected humeral head was compacted around the proximal portion of the stem to avoid an initial gap between the stem and the humerus. At the time of implant placement, patients with torsional instability were indicated for cement fixation. In the cemented fixation group, a vacuum mixing device^18^ and a cement gun were used.^29^ If the subscapularis tendon remained and could reach its original footprint, it was repaired using transosseous techniques. None of the patients underwent tendon transfer (eg, pectoralis major or latissimus dorsi transfer).

### Postoperative rehabilitation

All patients wore a shoulder abduction sling at 30° for 5 weeks. Active hand and wrist exercises were initiated immediately after surgery, and passive ROM exercises were initiated on postoperative day 4 under the supervision of physiotherapists. Active exercises in the supine and sitting positions were initiated 6 weeks postoperatively. Active rotational exercises were started 4 days postoperatively if the subscapularis tendon was nonrepairable, whereas passive and active rotational exercises were initiated 4 and 6 weeks after surgery, respectively, if the subscapularis tendon was repaired.

### Statistical analyses

Continuous variables (eg, ROM and age) and categorical variables (eg, sex, dominant hand involvement, and Hamada classification) were compared using the Mann-Whitney U test and Fisher’s exact test, respectively. A post-hoc analysis was performed to evaluate the validity of the power in CFR between the 2 groups. Preoperative and postoperative ROM and ASES scores were compared using the Wilcoxon signed-rank test. Inter-rater reliability was evaluated using Cohen’s kappa statistics. Statistical significance was set at *P* < .05. All statistical analyses were performed using the EZR software (Saitama Medical Center, Jichi Medical University, Saitama, Japan), which is a modified version of R commander (The R Foundation for Statistical Computing, Vienna, Austria). In addition, Cramer’s coefficients of association between CL and CNO and between CNO and CFR > 0.7 were also calculated.

## Results

### Baseline characteristics of the study participants

The baseline characteristics of the study participants are shown in Table I. No significant differences in age, dominant side, rotator cuff condition, and Hamada classification were identified among the study participants. However, the proportion of female patients was significantly higher in the cemented group.

**Table I:** Baseline characteristics of the cementless and the cemented groups.

	Cemetless (n = 74)	Cement (n = 35)	*P* value
Age, yr	79.2 ± 5.3	79.5 ± 5.8	.58
Dominant side	46 (62%)	24 (69%)	.67
Male	24 (32.4%)	3 (8.6%)	.0083∗∗
Condition of rotator cuffs	irreparable SSP, ISP tear intact SSC, Tm (n = 25)	irreparable SSP, ISP tear intact SSC, Tm (n = 15)	.85
	irreparable SSP, ISP tear repairable SSC, intact Tm (n = 16)	irreparable SSP, ISP tear repairable SSC, intact Tm (n = 6)	
	irreparable SSP, ISP, SSC tear intact Tm (n = 30)	irreparable SSP, ISP, SSC tear intact Tm (n = 13)	
	irreparable SSP, ISP, SSC Tm tear (n = 3)	irreparable SSP, ISP, SSC Tm tear (n = 1)	
Goutallier classification^20^	SSP: 3.8 ± 0.3	SSP: 3.6 ± 0.5	.016∗
	ISP: 3.5 ± 0.7	ISP: 3.2 ± 0.8	.051
	SSC: 2.9 ± 0.8	SSC: 2.6 ± 1.0	.22
	Tm: 0.9 ± 0.8	Tm: 0.7 ± 0.9	.65
Hamada classification	2 (n = 5)	2 (n = 9)	.062
	3 (n = 23)	3 (n = 12)	
	4a (n = 16)	4a (n = 6)	
	4b (n = 27)	4b (n = 7)	
	5 (n = 3)	5 (n = 1)	

*SSP*, supraspinatus; *ISP*, infraspinatus; *SSC*, subscapularis; *Tm*, teres minor.

Continuous variables are presented as mean ± standard deviation.

∗*P* < .05 and ∗∗*P* < .01.

### Comparison of preoperative and postoperative ROMs and ASES scores

Changes in the clinical outcomes of the cementless and cemented groups are shown in Table II. Significant differences were observed in shoulder flexion, shoulder abduction, and ASES scores. In contrast, no significant differences were observed in external and internal rotations. A comparison of the preoperative and postoperative clinical outcomes between the 2 groups is shown in Table III. No significant differences were found between the 2 groups.

**Table II:** Preoperative and postoperative changes in clinical outcomes for each group.

	Cementless (n = 74)		Cement (n = 35)	
	Preoperative	Postoperative	Preoperative	Postoperative
Flexion (°)	55 ± 23	132 ± 25	59 ± 19	128 ± 26
*P* value	*P* < .001∗∗		*P* < .001∗∗	
Abduction (°)	53 ± 20	129 ± 26	59 ± 17	125 ± 25
*P* value	*P* < .001∗∗		*P* < .001∗∗	
External rotation (°)	22 ± 18	25 ± 15	27 ± 16	27 ± 14
*P* value	*P* = .12		*P* = .89	
Internal rotation	6 ± 3 L3	6 ± 3. L3	7 ± 4 L2	7 ± 4 L2
*P* value	*P* = .53		*P* = .57	
ASES score	36.0 ± 6.8	81.2 ± 5.0	35.2 ± 6.4	78.4 ± 9.5
*P* value	*P* < .001∗∗		*P* < .001∗∗	

*ASES*, American Shoulder and Elbow Surgeons score; *L*, lumbar level.

Continuous variables are presented as mean ± standard deviation.

∗*P* < .05 and ∗∗*P* < .01.

**Table III:** Comparison of preoperative and postoperative range of motion between the 2 groups.

	Preoperative		Postoperative	
	Cementless (n = 74)	Cement (n = 35)	Cementless (n = 74)	Cement (n = 35)
Flexion (°)	55 ± 23	59 ± 19	132 ± 25	128 ± 26
*P* value	*P* = .21		*P* = .49	
Abduction (°)	53 ± 20	59 ± 17	129 ± 26	125 ± 25
*P* value	*P* = .092		*P* = .43	
External rotation (°)	22 ± 18	27 ± 16	25 ± 15	27 ± 14
*P* value	*P* = .16		*P* = .99	
Internal rotation	6 ± 3 L3	7 ± 4 L2	6 ± 3 L3	7 ± 4 L2
*P* value	*P* = .38		*P* = .39	
ASES score	36.0 ± 6.8	35.2 ± 6.4	81.2 ± 5.0	78.4 ± 9.5
*P* value	*P* = .54		*P* = .36	

*ASES*, American Shoulder and Elbow Surgeons; *L*, lumbar level.

Continuous variables are presented as mean ± standard deviation.

∗*P* < .05, ∗∗*P* < .01.

### Radiographic analyses using tomosynthesis in the cementless and cemented groups

**Figure 6:**
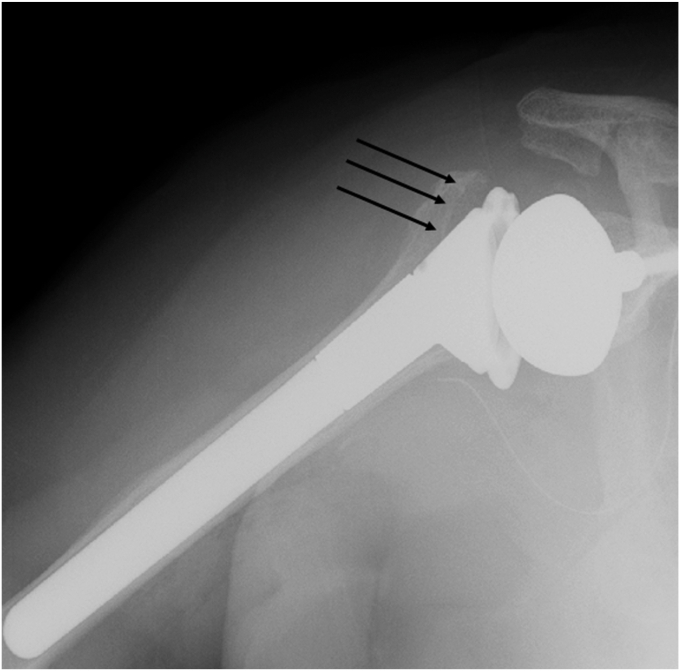
A radiolucent zone on the lateral side of the humerus on immediate postoperative radiographs. The medial portion of the stem did not fit into the medullary canal and was protruded.

Radiographic analyses using plain radiographs and tomosynthesis for both groups are shown in Table IV. Significant differences were observed in both the proximal and distal parts of the CFR between the cementless and cemented groups. However, no significant differences were observed in the incidence of OL or CNO between the 2 groups. OL was observed only in zone 7. No high-grade (≥ grade 3) CNO was observed in the cemented group. The Cramer’s coefficient of association between CL and CNO was 0.23, and a weak correlation was observed between the 2. All the patients with CNO were female. Six of the 7 patients in the cementless group with CNO showed a radiolucent zone on the lateral side of the humerus on immediate postoperative radiographs. The medial portion of the stem did not fit into the medullary canal and was protruding (Fig. 6). No prosthesis migration (stem sinking or valgus and varus angulation > 5°) or stem loosening was observed in either group (Cohen’s kappa coefficient was 1.0). No prostheses were fixed in varus or valgus angulations > 5° in either group.

**Table IV:** Radigraphic analyses using tomosynthesis in cementless and cemented groups.

	Cementless group (n = 74)	Cemented group (n = 35)
Canal filling ratio, % (proximal)	72.3 ± 9.9 (range: 45.1-88.5)	68.3 ± 11.1 (range: 44.5-91.0)
*P* value	*P* = .048∗	
Canal filling ratio, % (distal)	80.3 ± 11.0 (range: 55.3-99.0)	69.1 ± 11.3 (range: 56.1-99.0)
*P* value	*P* < .001∗∗	
Varus or valgus tilt relative to the bone axis > 5°	0 case	0 case
	*P* = 1.0	
Prosthesis migration (stem sinking, or valgus and varus inclination > 5°)	0 case (0%)	0 case (0%)
*P* value	*P* = 1.0	
Stem loosening	0 case (0%)	0 case (0%)
*P* value	*P* = 1.0	
Scapular notching	9 cases, grade 1	3 cases, grade 1
*P* value	*P* = .75	
Pedastal sign	0 case	
Condensation line	30 cases (zone 3: 1 case) (zone 4: 19 cases) (zone 3, 4: 4 cases) (zone 3, 4, 5: 6 cases)	
Osteolysis	5 cases (zone 7)	4 cases (zone 7)
*P* value	*P* = .46	
Osteopenia with cortical narrowing	7 cases (female: 7, male: 0) Mean age, yr: 75.7 (range 68-79) zone 1, grade 2 zone 2, grade 1 zone 1, grade 3 zone 1, 2, grade 3 zone 1, 2, grade 3 zone 2, grade 3 zone 1, 2, grade 3, zone 7, grade 2	3 cases (female 3, male 0) Mean age, yr: 74.0 (range 71-77) zone 1, grade 2 zone 1, grade 2 zone 1, 2, grade 1
*P* value	*P* = 1.0	
Complications	Acromion fracture^27^: 3 case (type I: 1, type II: 2) Dislocation: 1 case	Acromion fracture: 1 cases (type I: 1) Dislocation: 1 case
*P* value	*P* = .81	

Continuous variables are presented as mean ± standard deviation.

∗*P* < .05 and ∗∗*P* < .01.

### Comparison between groups with and without CNO

Comparisons between groups with and without CNO are presented in Table V. No significant differences in age, rotator cuff condition, Hamada classification, CFR at the proximal third, dCFR, or ROMs were observed between the 2 groups. However, post-hoc analysis showed that the power of this comparison was insufficient because of the small sample size in the CNO group (power: 0.38); therefore, a type II error should be considered. The CFRs tended to be larger in the CNO group.

**Table V:** Comparison with and without CNO groups.

	No CNO group 67 cases	CNO group 7 cases	*P* value
Sub zones of spot welds	Sub zone 1: 52 cases (78%)	Sub zone 1: 0 case (0%)	
	2: 47 cases (70%)	2: 1 case (14%)	
	3: 30 cases (45%)	3: 6 cases (86%)	
	4: 60 cases (90%)	4: 5 cases (71%)	
Age, yr	79.1 ± 5.3	76.9 ± 5.3	.22
Condition of rotator cuffs	irreparable SSP, ISP tear intact SSC, Tm (n = 23)	irreparable SSP, ISP tear intact SSC, Tm (n = 2)	.84
	irreparable SSP, ISP tear repairable SSC, intact Tm (n = 15)	irreparable SSP, ISP tear repairable SSC, intact Tm (n = 1)	
	irreparable SSP, ISP, SSC tear intact Tm (n = 26)	irreparable SSP, ISP, SSC tear intact Tm (n = 4)	
	irreparable SSP, ISP, SSC Tm tear (n = 3)	irreparable SSP, ISP, SSC Tm tear (n = 0)	
Hamada classification	2 (n = 5)	2 (n = 0)	1.0
	3 (n = 21)	3 (n = 2)	
	4a (n = 14)	4a (n = 2)	
	4b (n = 24)	4b (n = 3)	
	5 (n = 3)	5 (n = 0)	
pCFR	71.8 ± 10.1	75.6 ± 6.9	.35
dCFR	79.7 ± 11.1	85.9 ± 8.8	.14
Flexion(°)	134 ± 22	131 ± 29	.73
Abduction(°)	131 ± 23	125 ± 32	.49
External rotation(°)	26 ± 15	21 ± 22	.36
Internal rotation	6 ± 3	6 ± 4	.77
ASES score	81.2 ± 5.0	80.9 ± 9.0	.74

*SSP*, supraspinatus; *ISP*, infraspinatus; *SSC*, subscapularis; *Tm*, teres minor; *CNO*, osteopenia with cortical narrowing; *pCFR*, canal filling ratio at proximal thirds of prosthesis; *dCFR*, canal filling ratio at distal thirds of prosthesis; *ASES*, American Shoulder and Elbow Surgeons.

Continuous variables are presented as mean ± standard deviation.

The location of SW in the subzone analysis using tomosynthesis showed that SW was less frequently detected on the lateral side of the CNO group (subzones 1 and 2).

### Association between CNO and CFR > 0.7

The association between CNO and CFR > 0.7, and as an informative comparison, the association between CNO and CFR > 0.8 is presented in Table VI. The Cramer’s coefficients of association were 0.14 (*P* = .59) and 0.0025 (*P* = 1.0), respectively, and few correlations were observed.

**Table VI:** Association between CNO and CFR > 0.7 or > 0.8.

dCFR	CNO > grade 1	CNO (−)	*P* value
0.70 >	0	11	.59
0.70 <	7	56	
Cramer’s coefficient of association: 0.14			
dCFR	CNO > grade 1	CNO (−)	*P* value
0.80 >	3	29	1.0
0.80 <	4	38	
Cramer’s coefficient of association: 0.0025			

*dCFR*, canal filling ratio at distal thirds of prosthesis; *CNO*, osteopenia with cortical narrowing; *CFR*, canal filling ratio.

## Discussion

The results of this study support the hypothesis that cementless RTSA using the 10.13039/501100010693TM stem would have a similarly low incidence of CNO (7/74 cases, 9.5%) as cemented fixation. In the present study, CFR > 0.7 was not associated with the incidence of CNO (*P* = .59), and Cramer’s coefficient of association was 0.14. In contrast to the no-CNO group, the SW was less frequently detected in the lateral parts of the humerus in the CNO group (subzone 1, 78% vs. 0%; subzone 2, 70% vs. 14%).

The TM stem is characterized by proximal fixation, standard stem length, a conical proximal shape with an ovoid cross-section, and an in-growth–type (trabecular metal) surface coating. Female,^26^ distal (diaphyseal) fixation,^23^ long stems,^9^^,^^14^^,^^37^^,^^42^^,^^45^ press-fit or on-growth–type stem coating,^10^^,^^23^^,^^36^^,^^44^ and high CFR were risk factors for CNO after RTSA.^14^^,^^25^^,^^35^^,^^41^^,^^42^ All 7 patients who had CNO in the cementless group were female, which suggests that osteoporosis may influence the incidence of CNO.^26^^,^^41^ Several previous studies reported that CNO occurs more frequently in cementless fixation than in cemented,^7^^,^^41^ with an incidence of 10%-100%.^7^^,^^26^^,^^32^^,^^34^^,^^44^^,^^45^ In our study, there were 7 cases (9.5%) of CNO in the cementless group and 3 cases (8.6%) in the cemented group. There were no significant differences in the incidence of CNO between the 2 groups, and the incidence of CNO in the cementless group was lower than that reported in previous studies. Stress shielding characterized as the adaptation to the stress distribution has been well evaluated in hip arthroplasty,^13^^,^^56^ suggesting that long stem and high CFR provide stress reduction in the proximal part of the bone leading to bone resorption even in shoulder arthroplasty.^5^^,^^9^^,^^36^ In our study, patients with CNO had high CFR both in the proximal and distal parts of the humerus but not statistically significant. Moreover, CFR > 0.7 or > 0.8 were not associated with the incidence of CNO (Cramer’s coefficient of association: 0.14 and 0.0025, respectively), which did not support the results of previous studies indicating that CFR > 0.8 for RTSA increases the rate of CNO.^7^^,^^42^ A finite element study reported that bone stress changes were mainly associated with implant design (cross-sectional shape) rather than implant size, and demonstrated that oval and semi-angular stem designs reproduced native bone stress distribution.^2^ In addition, several previous studies reported satisfactory outcomes using oval stems.^35^^,^^37^^,^^45^ This suggests that the ovoid shape of the proximal part of the stem provides adequate stress distribution around the stem. A previous study compared 2 different stem designs (noncurved vs. curved stem) and demonstrated that the incidence of bone adaptation change was significantly higher in curved stems than in noncurved stems, although the CFR was higher in the noncurved stem.^10^ Likewise, the valgus-varus alignment indicated that the curved stem may affect bone stress.^54^ These results indicate that stem design is more important than size for preventing CNO. Despite the high CFR observed in the present study, the incidence of CNO was low. The TM stem, with its oval cross-sectional shape and noncurved design, may result in a low incidence of CNO.

**Figure 7:**
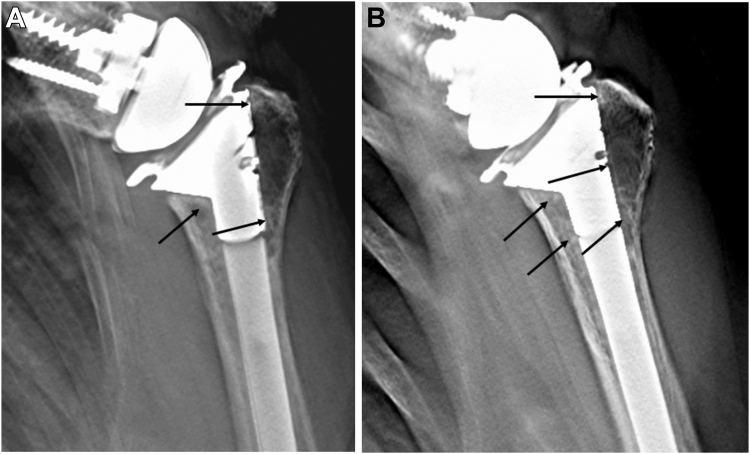
(**A**) Slight SW was recognized in subzones 1, 2, and 4 at 3 months postoperatively. (**B**) Clear SW was observed in subzones 1-4 at 6 months postoperatively. *Arrow*: spot welds. *SW*, spot welds.

An experimental study found that CT was unable to accurately detect a simulated bone graft resorption gap after increased bony offset RTSA (sensitivity, 38%; accuracy, 46%). Additionally, the bone resorption gap was underestimated.^15^ This result suggests that CT cannot detect implant loosening or bone incorporation due to metal artifacts. In contrast, previous studies demonstrated that tomosynthesis detected more radiolucent lines around the metallic implant than CT and plain radiography.^39^^,^^52^ Additionally, compared with plain radiography and CT, tomosynthesis improved the diagnostic accuracy in detecting signs of bone incorporation (ie, SW) in hip arthroplasty.^21^^,^^38^^,^^53^ A previous study using tomosynthesis suggested that biological fixation (SW) appeared within 3 months postsurgery.^39^ A representative case in our study demonstrated that slight SW was recognized in subzones 1, 2, and 4 (Fig. 7*A*) at 3 months postoperatively. Six months after surgery, clear SW was observed in subzones 1-4 (Fig. 7*B*). The Trabecular Metal Reverse Shoulder System (Zimmer Biomet, Warsaw, IN, USA) possesses material properties similar to those of trabecular bones as well as a surface coating conductive to bone in-growth.^4^^,^^28^^,^^33^

**Figure 8:**
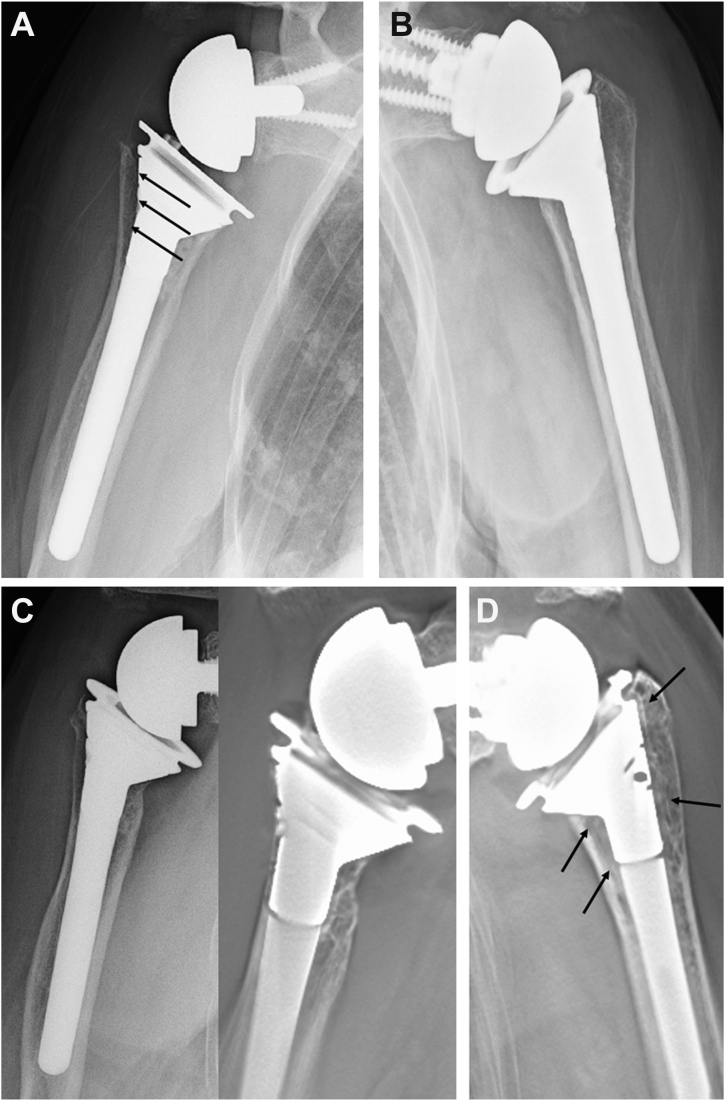
(**A**) The right shoulder (81-year-old female). The stem protruded from the medial calcar and subzones 1 and 2 had a radiolucent zone (initial gap) immediately after surgery. *Arrow*: radiolucent zone. (**B**) The left shoulder underwent reverse total shoulder arthroplasty 2 years after the right shoulder surgery. The stem did not protrude from the canal and no radiolucent zone was seen on plain radiograph immediately after surgery. (**C**) On the right shoulder, tomosynthesis at 1 year postoperatively demonstrated that osteopenia with cortical narrowing was observed in zones 1, 2 (grade 3), and 3 (grade 2), and no spot welds were observed in subzone 1 and 2. (**D**) On the left shoulder, spot welds were observed in subzones 1, 2, 3, and 4, and no bone resorption was observed. *Arrow*: spot welds.

The location of SW in the subzone analysis using tomosynthesis showed that the SW was less frequently recognized on the lateral side in the CNO group (subzones 1 and 2). In addition, 6 of the 7 patients in the cementless group with CNO had a radiolucent zone on the lateral side of the humerus, and the medial portion of the stem did not fit into the medullary canal and protruded. It is unclear whether CNO occurred because SW did not occur, or whether SW did not occur because CNO occurred. A patient who underwent RTSA in both shoulders using the same stem size is shown in Fig. 8. The right shoulder underwent a greater amount of osteotomy because of the marked upward migration of the humeral head. Consequently, the stem protruded from the medial calcar. In addition, zone 1 (subzones 1 and 2) was radiolucent (initial gap) immediately after surgery (Fig. 8*A*). The left shoulder underwent RTSA 2 years after the surgery on the right shoulder. In contrast to the right shoulder, the stem did not protrude from the canal and no radiolucent zone was observed on plain radiography immediately after surgery (Fig. 8*B*). Tomosynthesis at 1 year postoperatively shows that CNO was observed in zones 1 and 2 (grade 3) and 3 (grade 2) on the right shoulder, and no SW was observed in subzones 1 and 2 (Fig. 8*C*). In contrast, SW was observed in subzones 1, 2, 3, and 4, and no change in bone resorption was observed in the left shoulder (Fig. 8*D*). An in vitro study reported that the trabecular metal has more than twice the interfacial strength of the existing porous in 4 weeks and a high degree of bone ingrowth.^3^ As shown in Fig. 7, SW was observed on tomosynthesis at 3 months postoperatively. On the other hand, it is known that stress transfer to the cancellous bone is unevenly distributed depending on the location of the stem.^19^ In our study, patients with CNO had an initial gap on the lateral side of the humerus, which may have contributed to the uneven stress distribution, resulting in stress shielding (ie, CNO) in zones 1 and 2. Bone grafting at the time of stem insertion may be important not only to fill the initial gap but also to improve initial fixation strength.^8^ The reason for the lateral radiolucent zone and the protrusion at the medial portion may be due to the conical shape of the proximal portion of the TM stem. Owing to its conical shape, the stem does not fit into the medullary canal in small females or when excessive osteotomies are performed, requiring reaming of the calcar portion. In doing so, technical errors may result in the over-reaming of the lateral portion of the humerus. Other implant options may be appropriate for small women or patients who require excessive osteotomy.

Long stems are considered to have a higher frequency of CNO occurrence than short stems.^9^^,^^14^^,^^37^^,^^42^^,^^45^ Although the TM stem has only the traditional length (> 100 mm), the incidence of CNO is low. However, considering the results of previous finite elements study^43^^,^^49^ and bone stock preservation, a shorter stem would be ideal.

Considering that the CNOs observed in the cemented group were all low grade (< grade 2) and that there was no radiolucent area > 1 mm around zones 1 or 2, CNO in the cemented group may not be due to stress shielding, but rather to cement-induced thermal necrosis.^16^^,^^46^ OL was considered rare in the setting of hemiarthroplasty.^41^ As reported by previous studies, OL may be associated with cement fixation or polyethylene wear,^22^^,^^24^ especially with the scapular notch.^1^^,^^6^^,^^30^^,^^37^ In our study, 7 (cemented: 3 cases, cementless: 4 cases) of 9 patients with OL in zone 7 had low-grade scapular notching, which supports the results of previous studies.^1^^,^^37^

This study has some limitations. First, this was a retrospective study in which substantial selection bias (cemented or cementless) should be considered. Patients who underwent cement fixation, with a high proportion of females, may have had poor bone quality. Therefore, this comparative study had a potential selection bias.

Second, as the number of patients who developed CNO was small, it was necessary to consider type 2 errors in the statistical analyses. Third, tomosynthesis was performed on all patients, at least at the last follow-up; however, only a limited number of patients underwent tomosynthesis over time, with temporal changes observed. TM stems were cemented in this study. Cemented stems (like polished stems) should have been used. Although no adverse events occurred, the possibility of harm and potential adverse effects should be considered. Nevertheless, to our knowledge, this is the first study to investigate the radiographic change around the humeral stem using tomosynthesis that had clinical relevance.

## Conclusion

Cementless RTSA with the TM stem had a similarly low incidence of CNO as cemented fixation, and the incidence of CNO with the TM stem was lower than that reported in previous studies. CFR > 0.7 or > 0.8 was not associated with the incidence of CNO; however, the lack of SW at the lateral aspect of the humerus may have affected the incidence of CNO.

## Disclaimers:

Funding: No funding was disclosed by the authors.

Conflicts of interest: The authors, their immediate family, and any research foundation with which they are affiliated did not receive any financial payments or other benefits from any commercial entity related to the subject of this article.
